# A comparative review on heart ion channels, action potentials and electrocardiogram in rodents and human: extrapolation of experimental insights to clinic

**DOI:** 10.1186/s42826-021-00102-3

**Published:** 2021-09-08

**Authors:** Siyavash Joukar

**Affiliations:** 1 Cardiovascular Research Center, Institute of Basic and Clinical Physiology Sciences, Kerman University of Medical Sciences, Kerman, Iran; 2 Department of Physiology and Pharmacology, Afzalipour Faculty of Medicine, School of Medicine, Kerman University of Medical Sciences, P.O.Box 7616914115, Kerman, Iran; 3 Physiology Research Center, Institute of Basic and Clinical Physiology Sciences, Kerman University of Medical Sciences, Kerman, Iran

**Keywords:** Human, Rodents, Heart ion channels, Action potential, ECG

## Abstract

Electrocardiogram (ECG) is a non-invasive valuable diagnostic tool that is used in clinics for investigation and monitoring of heart electrical rhythm/conduction, ischemia/injury of heart, electrolyte disturbances and agents/drugs induced cardiac toxicity. Nowadays using animal models to study heart diseases such as electrical and mechanical disturbance is common. In addition, given to ethical consideration and availability, the use of small rodents has been a top priority for cardiovascular researchers. However, extrapolation of experimental findings from the lab to the clinic needs sufficient basic knowledge of similarities and differences between heart action potential and ECG of rodents and humans in normal and disease conditions. This review compares types of human action potentials, the dominant ion currents during action potential phases, alteration in ion channels activities in channelopathies-induced arrhythmias and the ECG appearance of mouse, rat, guinea pig, rabbit and human. Also, it briefly discusses the responsiveness and alterations in ECG following some interventions such as cardiac injury and arrhythmia induction. Overall, it provides a roadmap for researchers in selecting the best animal model/species whose studies results can be translated into clinical practice. In addition, this study will also be useful to biologists, physiologists, pharmacologists, veterinarians and physicians working in the fields of comparative physiology, pharmacology, toxicology and diseases.

## Background

Cardiovascular diseases (CVDs) are known as the most cause of death and it is estimated that deaths due to CVDs will increase more than 24 million a year by 2030 [1, 2]. Cardiac arrhythmias are responsible for at least half of sudden cardiac arrests [3]. Each irregular rate or rhythm in the electrical activity of the heart is known as arrhythmia. Much of the information available on the electrical activity of excitable cells, including muscle cells and conduction system of the heart, has been obtained through experimental studies on animal specimens. Selection of appropriate animals and models in cardiac electrophysiology research not only benefit to discover antiarrhythmic drugs, but also important to study the normal function of ion channels, arrhythmias caused by channelopathy, to investigate the safety of drugs and aptitude tests for arrhythmias. Obviously, in order to generalize the results of the research to humans, researchers should be aware of the limitations and benefits of any animal model and point out when publishing their studies. Although some limitations for different species and models are unavoidable, the models/species must still be approved for a specific study. For example, a model may be limited in ease of use or cost, while being validated. When the model/species are not fully validated for a study, it is desirable to provide information on different species and models to reduce the risk of generalizing the results of that species/model to humans. Overall, selection of a suitable species/model should reflect symmetry between expectation of obtaining analyzable data and expectation of obtaining data that rightly predicts clinical effects [4]. Mammals used for cardiovascular studies include goats, sheep, dogs, pigs, rabbits, guinea pigs, rats, and mice. Given the ethical considerations as well as the high costs of working on large mammals, nowadays, the use of laboratory rodents and their genetically modified models in biological studies and diseases, including cardiovascular diseases, is common in many research laboratories. In the present review by using existing evidences, the similarities and differences of electrocardiograms, the cardiac action potential and corresponding ion channels in humans and some important laboratory rodents were compared. To achieve this goal, all papers related to human and rodents’ ion channels, action potentials and electrocardiogram, published from 1980 by the end of 2020 in different electronic databases such as Web of Science, Google Scholar, PubMed, Science Direct, EMBASE, Springer Link, Scopus, Biological Abstracts, DOAJ, ISC and Chemical Abstracts were collected and evaluated and then the elite of information was summarized in this work. Free-text terms, and combination of relevant keywords were including: human, rodents, rabbit, guinea pig, rat, mouse, heart, conductive system, electrocardiogram (ECG), ECG waves, ST segment, QT interval, PR interval, action potential, depolarization, repolarization, ion channels, resting membrane potential, cardiac arrhythmia, sinoatrial node, atrioventricular node, purkinje fibers, heart rate variability (HRV), cardiac injury, myocardial infarction, arrhythmia and so in.

This review provides a platform that can help researchers to identify the best species/model to study the electrical activity of the heart in physiological and pathological conditions including the investigation of ion channels behavior, electrical heart disorders, induction and study of various arrhythmias, and also study the safety, antiarrhythmic and pro-arrhythmic effects of drugs, agents and toxins.

## Main text

### Heart electrical activity

#### Excitatory and conduction system

**Fig. 1:**
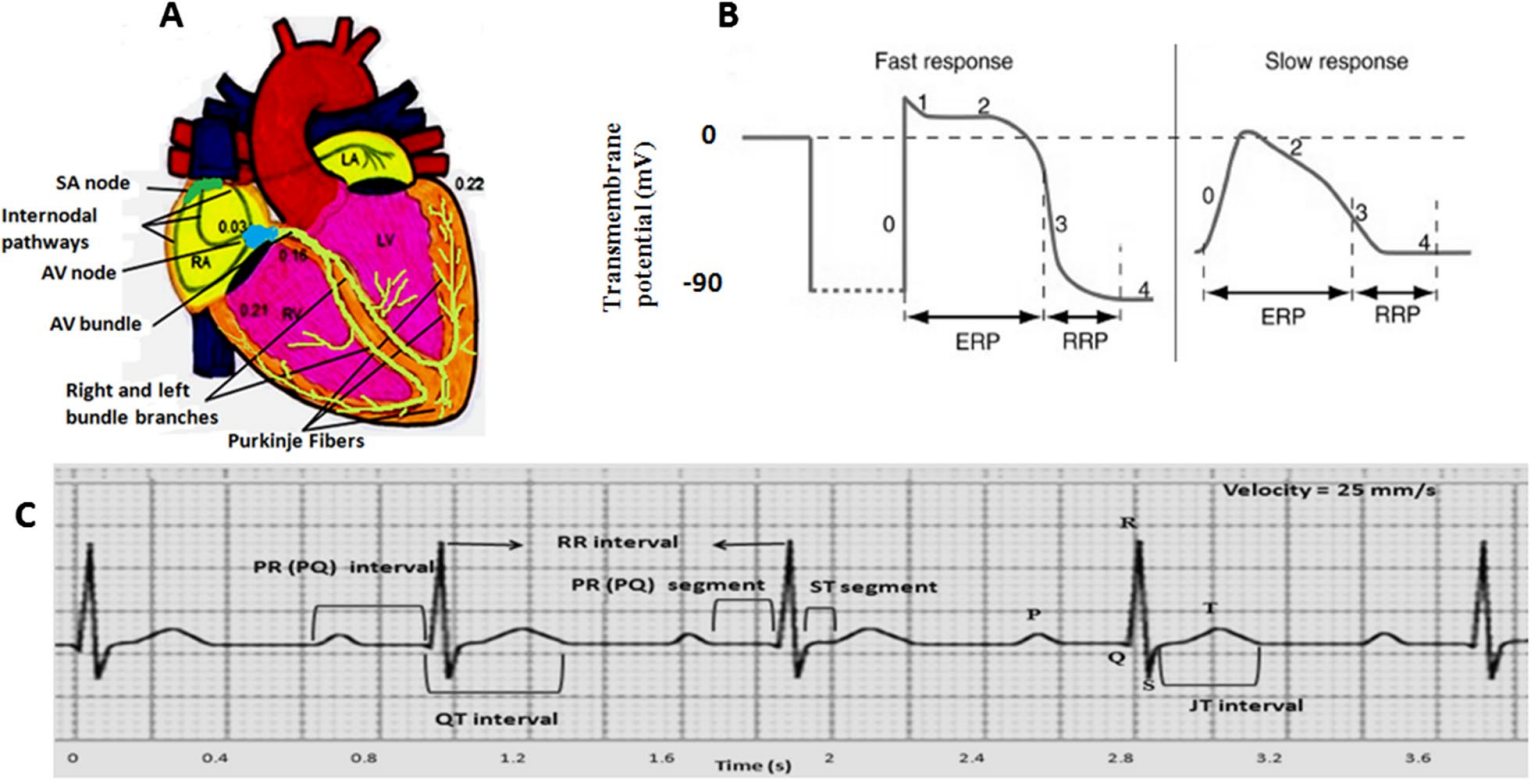
**A** Schematic representation of the excitatory and conductive system of the heart and the time of impulse appearance (In hundredths of a second after starting at the SA node) in different parts of the heart. SA; sinoatrial, AV; atrioventricular, RA; right atrial, LA; left atrial, RV; right ventricle, LV; left ventricle. **B** cardiac fast and slow action potentials and different phases. **C** A normal electrocardiogram where P and T waves, QRS complex, intervals and segments are shown. ERP; effective refractory period, RRP; relative refractory period

The heart has an excitatory-conduction system that spontaneously and regularly generates electrical impulses (action potentials) and spreads throughout the heart (Fig. 1A). Excitatory and conductive system includes sinoatrial (SA) node, internodal pathways, atrioventricular (AV) node, the bundle of His, right and left bundle branches, and Purkinje fibers [5, 6]. Under normal conditions, the SA node is the dominant heart pacemaker because of having the fastest firing rate. Shortly after initiating in the SA node (about 0.03 s), the electrical impulse reaches the AV node through the internodal pathways. In physiological conditions, the only way for the conduction of impulses from the atria to the ventricles is the AV node-His bundle pathway. The AV node conducts the electrical impulses to the bundle of His with low velocity (it lasts about 0.13 s). Naturally, it takes 0.12 to 0.20 s for the impulse to reaches the ventricles after starting at the SA node. This delay allows the atria to contract sooner than the ventricles and provides enough time for optimal ventricular filling. For impulse to depolarize all of the ventricular myocytes, it takes 0.06 to 0.1 s. Low conduction time in the ventricles allowing the ventricular cells to have a relatively coordinated contraction [5–8].

#### Cardiac action potentials

There are two types of action potential (AP) in the heart. Slow/brief action potential that is created in the SA and AV nodes, and fast/long action potential that occurs in the atrial and ventricular myocytes and the specialized conducting fibers (Fig. 1B) [8]. A fast action potential has several phases. Phase 0 is a rapid initial depolarization which due to the opening voltage-dependent Na^+^ channels and the entry of Na^+^ ions into the cell. During this phase, the membrane potential from negative values of about − 90 millivolt (mV) reaches to positive values of about + 20 mV. Phase 1 shows the initial and brief repolarization caused by potassium efflux through transient outward K^+^ channels and by the inactivation of the Na^+^ channels. In phase 2 or plateau, calcium ions enter the cell through L-type Ca^2+^ channels, however, the influx of Ca^2+^ ions is approximately equal to the efflux of K^+^ ions that leave the cell through various K^+^ channels. Therefore, the membrane potential remains almost constant. The plateau phase gives the ventricles time to have an optimal contraction. Phase 3 is indicative of final repolarization and during it, the membrane potential returns to resting level. This phase in ventricular cells corresponds to T wave on the electrocardiogram (ECG) (Fig. 1C). Different K^+^ currents are responsible for phase 3 such as rapid and slow delayed rectifier K^+^ currents (I_Kr_ and I_Ks_), inwardly rectifier K^+^ current (I_K1_) and transient outward K^+^ current (I_to_). From the onset of the action potential until part of phase 3, the cell does not respond to new excitation, which is called the effective (or absolute) refractory period (ERP or ARP). Phase 4 represents the resting membrane potential, where again potassium currents are the main determinants, especially I_K1_ [5, 7–9]. In slow action potential, phase 0 has fewer slope and amplitude, Phase 1 does not exist, phase 2 is shorter and not flat, as well as resting membrane potential, phase 4, is less negative and is not constant so that a slow diastolic depolarization occurred during this phase results in the spontaneous and rhythmic activity of the heart. The generation of diastolic depolarization is mediated by different channels. One of the most important channels is a member of the family of hyperpolarization-activated cyclic nucleotide-gated (HCN) channels. These channels are activated in the late phase of repolarization at the potential of about − 60 mV and carry an inward current named funny current (I_f_). Under physiologic conditions, the funny current consists of mainly Na^+^ and slightly Ca^2+^. In addition, it is suggested other ionic currents through T-type Ca channels, Na^+^/Ca^++^ exchanger, sustained inward Na^+^ current are present in SA node cells along with transient receptor potential (TRP) channels which can involve in diastolic depolarization. In this type of action potential, phase 0 is mainly caused by calcium influx via L-type Ca^2+^ channels [5, 8, 10].

### Electrocardiogram (ECG)

ECG is the average of instantaneous electrical activities of all excitable cells and not any individual cell at any given time in the heart, which is recorded by electrodes from the body surface (Fig. 1C). The ECG contains a few deflections and intervals. The P wave, the QRS complex, and the T wave exhibit the atrial depolarization, the ventricular depolarization, and the ventricular repolarization, respectively. PR interval (from the onset of the P to the onset of the Q or R) is the required time to the impulse reaches from the SA node to the ventricles. The QT interval (from the onset of the Q or R to the end of the T) is the required time for ventricular depolarization and repolarization. The ST segment (from the end of the S to the onset of the T) corresponds to the plateau phase in ventricular myocytes [6, 11]. ECG reflects the spatiotemporal distribution of extracellular voltage of the heart. Therefore, we are able to infer changes in cardiac electrical properties based on changes in the electrocardiogram and hence rhythm and conduction disturbances, heart anatomical orientation, the presence of coronary insufficiency as cardiac ischemia, injury or necrosis, alteration in electrolyte concentrations, the effects of some drugs, diseases and nutritional deficiencies. ECG indirectly shows the evidence of structural cardiac muscle and structural remodeling that in turn can affect automaticity and impulse propagation of electrical impulse. While, ECG does not measure contraction of the cardiac muscle, however, it reflects instantaneous changes in cardiac cell membrane or channels, or intracellular signaling and ion regulation that stem from the status of heart biochemical and metabolic processes [12]. In addition, ECG is used to estimate heart rate variability (HRV), the degree of difference in the interbeat intervals of successive heartbeats, an indicator of the heart sympathovagal balance [13, 14]. To assess ECG rhythm in humans, analysis of rate, regularity of waves sequence, PR interval, QRS interval, QT interval, waves’ voltage and duration, ST shift, dropped beats or pauses and groupings of QRS complexes and so in, should be considered.

### Mechanisms of cardiac arrhythmias

Briefly, the mechanisms causing cardiac arrhythmias can be classified into two categories, abnormal impulse generation, abnormal impulse propagation or a combination of both [15, 16]. Disorders of impulse generation may affect automaticity or create triggered activity. Different factors such as autonomic nervous system and metabolic abnormalities can alter normal automaticity and elicit sinus bradycardia or sinus tachycardia. Low HRV which reflects increased sympathetic tone can be associated with an increased risk of cardiac arrhythmia and mortality rate. In some circumstances such as elevated extracellular potassium, low intracellular pH, and catecholamine excess, non-pacemaker myocardial cells that in normal hearts have no spontaneous activity may begin to produce electrical impulses and cause abnormal automaticity [15]. Premature beats, atrial tachycardia, accelerated idioventricular rhythm and ventricular tachycardia may be the result of these ectopic foci activities. In the triggered activity, the impulse for myocardium depolarization is started by afterdepolarization. Afterdepolarization is oscillations in cell membrane potential that happen during or immediately after a preceding AP (trigger). When afterdepolarization potential reaches the threshold for stimulation of cells, a new AP is created. Based on the start time, 2 types of afterdepolarizations have been described: early afterdepolarizations (EADs) occur during AP plateau or late repolarization (phase 2 or 3 of the AP) and delayed afterdepolarizations (DADs) occur after completion of the repolarization (during phase 4) [8, 15]. Some predisposing factors for EAD creation include hypokalemia, hypoxia, and acidosis. EADs can result in torsade de pointes, a life-threading and polymorphic ventricular tachyarrhythmia. The variety of conditions such as toxic concentrations of digitalis which suppress the Na^+^/K^+^ pump and enhance the free intracellular calcium, catecholamines, hypokalemia, hypercalcemia, cardiac hypertrophy and heart failure, that boost the intracellular Ca^2+^ concentration during heart diastolic phase can cause DAD. During calcium overload, DAD especially is initiated when heart rate increases (decrease in cycle length). Increasing the amplitude of the DAD and reaching the membrane potential to the threshold can lead to triggered activity [8, 15, 17].

Disorders of impulse propagation include cardiac blocks and reentry. The cardiac blocks delay or stop the impulse conduction through the conductive system. Many factors such as some drugs or degenerative processes can develop the blocks. In re-entry disturbances, a group of myocardial cells that are not activated during the early stage of depolarization can resume excitability before the impulse vanishes. In this situation, they may act as a connection to re-excite zones that were previously depolarized but were recovered from the refractory period of the initial wave. The prolonged conduction time and shortened refractory period are two important re-entry predisposing factors. Re-entry is the most dominant mechanism of arrhythmias in the clinical setting and occurs due to anatomical and functional reasons [15].

### Comparison of cardiac structure and function in rodents and human

#### General structure and function

A heart-to-body weight ratio of rat is less or similar, guinea pig is similar and rabbit is less than human but in mouse is more than human [18, 19]. Structure of T-tubule openings (mouths) are more consistent in rat, rabbit, and human and are unobstructed, while mouse T-tubule mouth regions are narrow and convoluted [20]. Similar to humans, Purkinje fiber network in rabbits, rats and mice is limited to the sub-endocardium [21]. Rabbit and rat gap junctions’ distribution and intercalated disks architecture are similar to the human heart [22, 23]. Instead of superficial main coronary artery branches in humans, the main coronary artery branches of rat and mouse are embedded within the myocardium [3]. There is a close correlation between the volume densities of mitochondria, heart rate and oxygen consumption in man, dog, pig, rabbit, guinea pig, rat and mouse, so that the smaller animal has higher VO2 and higher mitochondrial content of the myocardium. However, the volume densities of myofibrils remain constant in all species [24]. Rat and mouse heart muscles show shorter actin-myosin cross-bridge attachment time with low contractile force, high shortening velocity and high heart rate in comparison with rabbit and human [25]. In addition, rat and mouse hearts show a negative staircase, however rabbit, guinea-pig and other larger mammals and human hearts show a positive staircase phenomenon, which increasing heart rate leads to increasing cardiac contractile force [26, 27]. Some study indicated that the force–frequency relation is bell-shaped, with a positive slope at low, non-physiological rates and a slightly negative slope over the physiological range in rat [28]. The rate of Ca^2+^‏ removal through Na^+^‏–Ca^2^‏^+^‏ exchanger is greater in hamster then followed by guinea pig, human, and rat, respectively [29].

#### Comparison of cardiac action potentials and ion channels in rodents and human

As mentioned above the unique type of the SA node action potential comes from the activities of a number of underlying ionic currents. These include the funny current (If) carried by hyperpolarization-activated cyclic-nucleotide gated (HCN) channels, T- and L-type Ca^2+^ currents, a sustained inward sodium current (Ist), a number of voltage-dependent K^+^ currents, and a Na^+^–Ca^2+^ exchange (NCX) current (INCX). The morphology of action potential and density of ion channels subtypes gradually changes from center to peripheral of node and also in atrium and ventricle of heart. Action potential propagated from center to neighboring cells through gap junctions. Connexins are the structural proteins of gap junctions. They have different subtypes and their densities are dissimilar in SA node than other parts of cardiac muscle. The diversity in currents and channel density lead to discrepancy in type, duration, amplitude and frequency of action potential in animal species. For example, it is believed that high density of ICaT in mouse SA node cells would accelerate pacemaker depolarization, thereby increasing heart rate [30]. The main ionic currents in center of SA node in human, rabbit, guinea pig, rat and mouse and also their roles in excitable activity are summarized in Table 1 [31–42].

**Table 1:** Main ionic currents and their proteins, gens, and function in cardiac SA node of human and small animals mediating excitable activity

Current/Transporter	Protein (α sub unit)	Gen	Ion	Direction	Function				
If	HCN1, HCN4	hHCN1, hHCN4	Na^+^	Inward	Involves in Diastolic depolarization	Yes, HCN1, HCN4	Yes, HCN1 > HCN4	Yes, HCN4 > HCN1	Yes, HCN4 > HCN2 > HCN1 in mouse [31, 32, 35, 36, 39–41]
*I*st	CaV1.3	CACNA1D	Na^+^	Inward	Involves in Diastolic depolarization	Yes?	Yes, CaV1.3	Yes, CaV1.3	Yes, CaV1.3 [31, 33, 34]
ICaT	Cav3.1, Cav3.3	CACNA1G, CACNA1I	Ca^2+^	inward	contributes to the final phase of diastolic depolarization	Yes, Cav3.1, Cav3.3	Yes, Cav3.1, Cav3.3	Yes, Cav3.1, Cav3.3	Yes, Cav3.1 > Cav3.2 [31, 33, 35, 36, 39]
ICaL	Cav1.3, Cav1.2	CACNA1D CACNA1C	Ca^2+^	Inward	Responsible for the SA node action potential upstroke	Yes, Cav1.3 > Cav1.2	Yes, Cav1.3 > Cav1.2	Yes, Cav1.3,Cav1.2	Yes, Cav1.2 > Cav1.3 [31, 33, 35, 36, 39, 41]
INCX	NCX1, NCX2	*SLC8A1*	Na^+^ , Ca^2+^	Inward/outward	contribute to the final phase of diastolic depolarization	Yes, NCX1, NCX2	Yes, NCX1, NCX2	Yes, NCX1, NCX2	Yes, NCX1, NCX2 [31, 35–39, 41]
IKr	Kv11.1	*KCNH2*	K^+^	Outward	Responsible for repolarization of the SAN AP	Yes, Kv11.1	Yes, ?	– ?	Yes, Kv11.1 [31, 35, 36, 39, 41]
IKs	Kv7.1	*KCNQ1*	K^+^	Outward	Responsible for repolarization of the SAN AP	Yes, Kv7.1	Yes, Kv7.1	Yes, Kv7.1	Yes Kv7.1 [31, 35, 36, 39]
IK1	Kir2.1/Kir2.2	*KCNJ2*	K^+^	Outward/inward	Responsible for end of repolarization of the SAN AP and Resting potential	Yes, Kir2.1/Kir2.2	Yes, Kir2.1	?	Yes, Kir3.4 [31, 35, 36, 39–41]
Connexins (Cx)	Cx45, Cx40, Cx43	*GJC1, GJA5, GJA1*	Non-specific ion channels	Bidirectional	Couples the neighboring cells as relatively weakly	Yes, Cx45, Cx40	Yes, Cx43, Cx45, Cx40	Yes, Cx43, Cx45, Cx40	Yes Cx45 [31, 35, 39, 41, 42]

INa, fast Na^+^ current; Ist, sustained inward Na^+^ current; IKur, ultra-rapid outward K^+^ current; ICaL, L-type Ca^2+^ current; IKr, rapid delayed rectifier K^+^ current; IKs, slow delayed rectifier K^+^ current; IK1, inward rectifier potassium current; INCX, Na^+^–Ca^2+^ exchanger current; NCX, Na^+^/Ca^2+^ exchanger protein; SAN, sinoatrial node; AP, action potential; ?, indistinctive

**Fig. 2:**
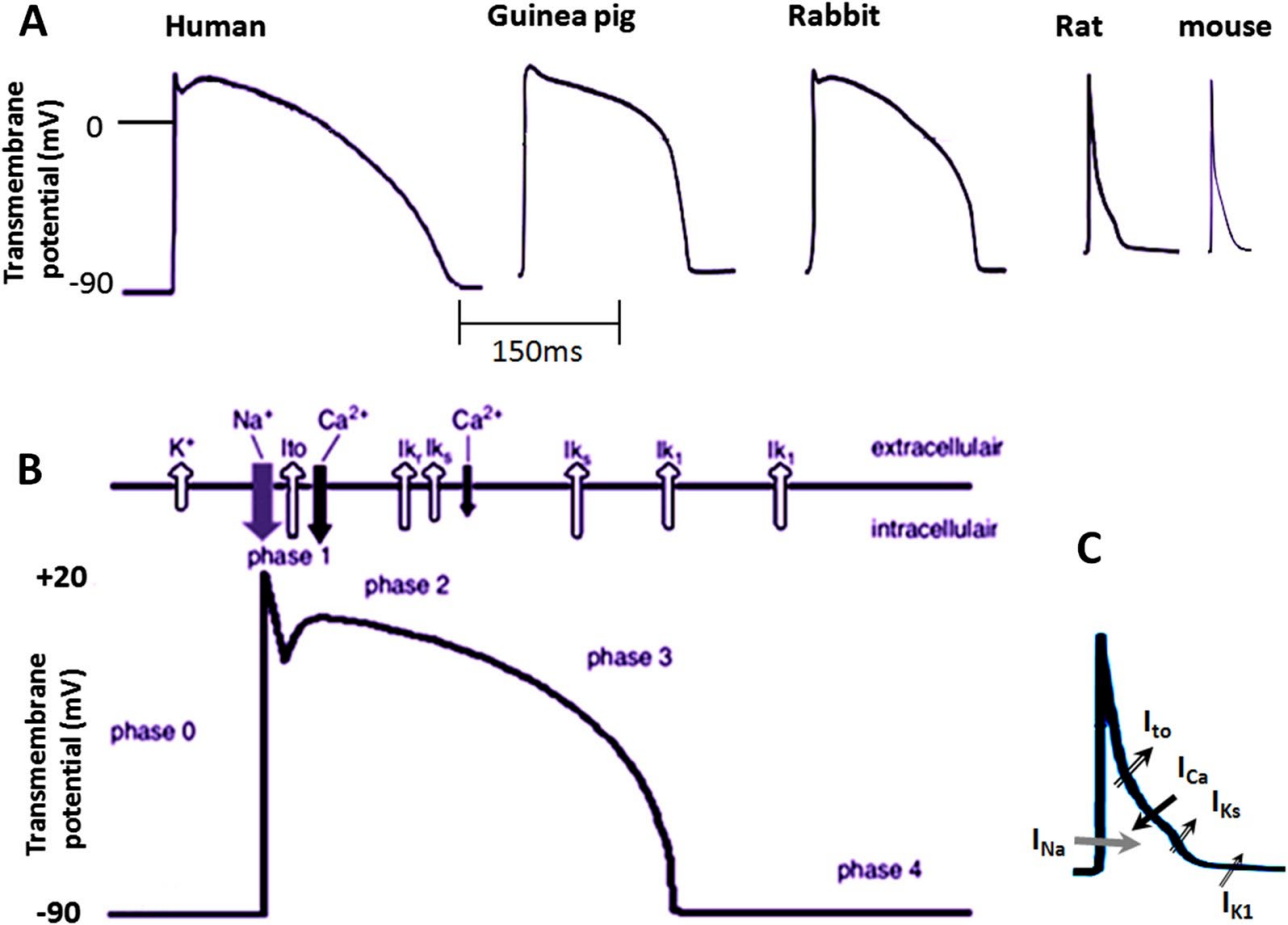
**A** Types of action potential of ventricular myocytes in human and rodents. **B** The main ion currents at different phase of the action potential in human. **C** The main ion currents at different phase of the action potential in rat and mouse. The direction of the arrows indicating the direction of ions flow

The important channel/current involving in action potential in mammalian heart atria and ventricles are including INa (Na^+^ inward current, phase 0, depolarization), Ito fast, (K^+^ outward current, phase1, repolarization), Ito slow (K^+^ outward current, phase1, 2, repolarization), IK ultra rapid (K^+^ outward current, phase1, 2, repolarization), IK rapid and IK slow (delayed rectifier K^+^ outward current, phase2, 3, repolarization), IK1 (:inwardly rectifier potassium K^+^ outward/inward current, phase3 repolarization and resting phase), ICa (Ca^2+^ inward current, phase2, repolarization) which depending on the species their density varies (Fig. 2B, C). The cardiac ion channels distribution and function show most similarity among dog, pig, rabbit and human. In their ventricles the IKr (rapidly activating delayed rectifier K‏ current) is the predominant repolarizing current. While, guinea pig ventricular repolarization relies on IKs (slowly activating delayed rectifier K‏ channel current) in both atrium and ventricles, rat and mouse ventricular repolarization rely on IKto (Ito) (transient outward K‏ channel current) and guinea pigs lack IKto [43]. Unlike humans, inhibition of IKr and IKs in murine has no significant effect on atrium and ventricular repolarization [44] and IKto and IKur has the most effect on ventricular repolarization (Fig. 2A–C, and Tables 2 and 3) [7, 19, 31, 45–49]

**Table 2:** Main ionic currents and their proteins, gens and densiti in cardiac atrium of human and small animals mediating excitable activity

Action potential phase	Current/transporter	Protein (α sub unit)	Gen					
0	INa (inward)	Nav1.5	*SCN5A*	+++	+++	+++	++++	++++ [7, 19, 31, 47, 48]
1	Ito,f (outward)	Kv4.2 Kv4.3	*KCND2 KCND3*	+++	+	–	+++	+++ [7, 31, 45, 46, 48]
1	Ito,s (outward)	Kv1.4	*KCNA4*	+++	+++	–	+++	+++ [7, 31, 45–48]
1	IKur (outward)	Kv2.1 Kv1.5	*(KCNB1) (KCNA5)*	++	++	+	++	++ [7, 31, 46]
2	ICaL (inward)	Cav 1.2	*CACNA1C*	++	++	++	++	++ [7, 31, 45, 47, 48]
2, 3	IKr (outward)	Kv11.1	*KCNH2 (HERG)*	+	+	+	+	+ [7, 19, 31, 45–48]
2, 3	IKs (outward)	Kv7.1 (KvLQT1)	*KCNQ1*	+	+	++	+	+ [7, 19, 31, 45–48]
3	IK,slow1 (outward)	Kv1.5	*KCNA5*	-	-	-	+	+ [7, 31, 45, 47, 48]
3	IK,slow2 (outward)	Kv2.1	*KCNB1*	–	–	–	+	+ [7, 31, 45, 48]
3	Iss (outward)	Kv1.5	*KCNA5*	–	–	–	+	+ [7, 31, 48]
3	Atrial-specific 4-aminopyridine-sensitive (outward)	Kv1.5	*KCNA5*	++	?	?	++	++ [7, 31, 45]
3 and 4 RMP	IK1 (outward)	Kir2.1/Kir2.2	*KCNJ2*	+	+	+	+	+ [7, 31, 45, 47, 48]
2	INCX	NCX1, NCX2	*SLC8A1*	+	+	+	+	+ [7, 47]

INa, fast Na current; IKur, ultra-rapid outward K current; Ito,f, fast transient outward K current; Ito,s, slow transient outward K^+^ current; ICaL, L-type Ca current; IKr, Rapid delayed rectifier K current; IKs, slow delayed rectifier K current; IK,slow1, 4-Aminopyridine-sensitiverapidly activating slowly inactivating K current1; IK,slow2, 4-Aminopyridine-sensitiverapidly activating slowly inactivating K current2; Iss, sustained 4-aminopyridinesensitive delayed rectifier K current; IK1, inward rectifier potassium current; INCX, Na^+^–Ca^2+^ exchanger current; RMP, resting membrane potential; – (no existing); + (the existing rate); ?: indistinctive

**Table 3:** Main ionic currents and their proteins, gens and density in cardiac ventricle of human and small animals mediating excitable activity

Action potential phase	Current/transporter	Protein (α sub unit)	Gen					
0	INa (inward)	Nav1.5	*SCN5A*	+++	+++	+++	++++	++++[7, 19, 31, 47–49]
1	Ito,f (outward)	Kv4.2 Kv4.3	*KCND2 KCND3*	++	+	–	+++	+++ [7, 31, 45, 46, 48]
1	Ito,s (outward)	Kv1.4	*KCNA4*	++	+	–	++	++ [7, 31, 45–49]
1	IKur (outward)	Kv2.1 Kv1.5	*(KCNB1) (KCNA5)*	–	+	–	++	++ [7, 31, 46, 49]
2	ICaL (inward)	Cav 1.2	*CACNA1C*	+++	+++	+++	++	++ [7, 31, 45, 47–49]
2, 3	IKr (outward)	Kv11.1	*KCNH2 (HERG)*	+++	+++	+++	+	+ [7, 19, 31, 45–49]
2, 3	IKs (outward)	Kv7.1 (KvLQT1)	*KCNQ1*	+++	+++	++++	+	+ [7, 19, 31, 45–49]
3	IK,slow1 (outward)	Kv1.5	*KCNA5*	–	–	–	++	++ [7, 31, 45, 47, 48]
3	IK,slow2 (outward)	Kv2.1	*KCNB1*	–	–	–	++	++ [7, 31, 45, 48]
3	Iss (outward)	Kv1.5	*KCNA5*	–	–	–	+	++ [7, 31, 48]
3 and 4 (RMP)	IK1 (outward)	Kir2.1/Kir2.2	*KCNJ2*	++	++	++	++	++ [7, 31, 45, 47–49]
2	INCX	NCX1, NCX2	*SLC8A1*	++	++	++	++	++ [7, 47, 49]

INa, fast Na current; IKur, ultra-rapid outward K current; Ito,f, fast transient outward K current; Ito,s, slow transient outward K^+^ current; ICaL, L-type Ca current; IKr, rapid delayed rectifier K current; IKs, slow delayed rectifier K current; IK,slow1, 4-aminopyridine-sensitiverapidly activating slowly inactivating K current1; IK,slow2, 4-aminopyridine-sensitiverapidly activating slowly inactivating K current2; Iss, sustained 4-aminopyridinesensitive delayed rectifier K current; INCX, Na^+^–Ca^2+^ exchanger current; RMP, resting membrane potential; – (no existing); + (the existing rate)

The difference of the IKto density and high magnitude of Ito in rat/mouse than human causes the rat and mouse hearts, action potentials have a large initial repolarization phase and a short/lack plateau phase at a lower membrane potential than human. In rabbits, depending on the regions of the ventricle from which the individual myocytes are derived, the action potential has or has not a rapid phase of repolarization and discernible Ito. However, it is reported that the action potential of guinea pig ventricular myocytes has not phase 1 and there has been no evidence of Ito. Both rabbit and guinea pig action potential have obvious plateau phase (Fig. 2A). Ventricular action potentials duration of rat and mouse are shorter and has a short/lack of the well-defined plateau phase at a lower membrane potential [46, 50]. Results obtained from previous studies confirmed that during action potential there is no difference between amplitude and voltage dependence of Ica and inactivation kinetics of Ica between rabbit, rat and guinea pig ventricular myocytes [50]. As mentioned above in rats the IK1 channel density is less than rabbit or guinea pig. During depolarization, although the amplitude of outward currents increased with more positive potential, the patterns are different. In rabbit the initial repolarization due to Ito is followed by the plateau of the action potential, and then the inactivation of L-type calcium current and finally the magnitude of IK1 increased, thus leading to final repolarization. In guinea pigs there is no Ito but IK is activated during the plateau and contributes to repolarization. So, the shape of the ventricular action potential is similar to rabbit however it has not phase 1 [50]. Similar to rabbit, human cardiac action potential demonstrates one noticeable notch in phase 1 repolarization, but has a more negative resting potential of ~ − 90 mV [51] compared with that rabbit ~ − 80 mV [52], guinea pig ~ − 80 mV [53], rat ~ − 70 mV [54] and mouse ~ − 73 mV [55]. However, various studies have reported different amounts of membrane resting potential in small animals, the approximate range of which is shown in Table 4. Apparently, this difference may come from the larger expression and density of Ik1 in human cardiomyocytes [56]. In human ventricle the main repolarization outward currents are including Ito fast (Ito1), Ito slow (Ito2), IKr, IKs, and IK1, respectively, and main repolarization inward current is ICa^2+^ in phase 2 (Fig. 2B). The main ionic currents responsible for action potential of atrium and ventricle of rodents and humans and their proteins/gens/density are compared and have been summarized in Tables 2 and 3 [7, 19, 31, 45–49].

**Table 4:** Comparison of maximum diastolic potential of SA node, resting membrane potential of ventricular muscle, heart rate and some ECG parameters between human and rodents

Mammal	Parameters					
			HR/min	PR interval/ms	QRS/ms	QTc/ms
	~ − 62	~ − 90	60–100	120–200	60–100	391–449, 459^b^ [58]
	~ − 65	range − 80–(− 90)	130–330	40–80	20–60	238–248 [59, 60]
	~ − 59	range − 80–(− 90)	192–338	55–78	53–75	264–265 [57, 65]
	~ − 58	range − 70–(− 80)	239–508	38–70	12–22	119–141^a^ [61, 64]
	~ − 58	range − 70–(− 80)	242–452	39–78	14–28	133–173 [61]
	~ − 60	range − 68–(− 80)	431–800	33.8–66.8	9.9–11.7	39.3–45.3 [62, 63]

MDA, maximum diastolic potential of SA node; RMP, resting membrane potential; HR, heart rate; QTc, QT interval corrected by Bazett’s formula

^a^QTc corrected by Fridericia formula

^b^449 for male and 459 for female based Framingham formula

^c^(Sprague–Dawley)

^d^(Wistar)

#### Comparison of ECG in rodents and human

**Fig. 3:**
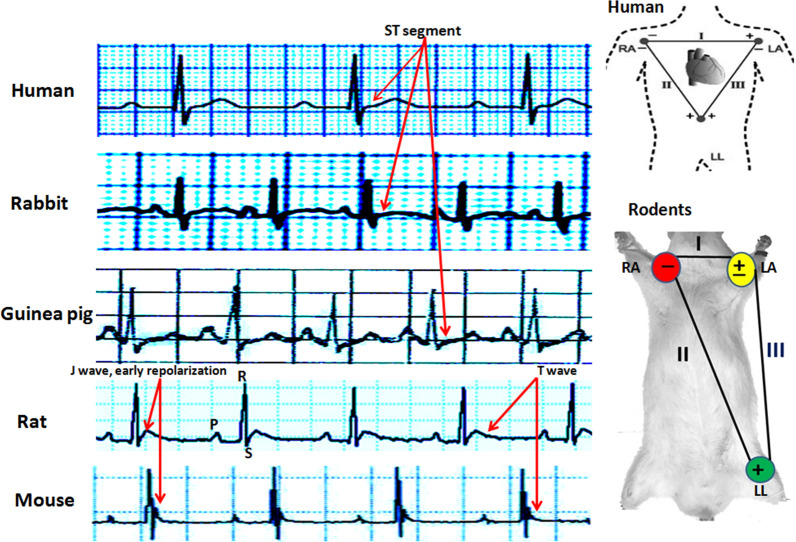
Strips of lead II of electrocardiogram in human and redents and also location of electrodes connection to record human and rodent limbs leads of electrocardiogram. Consider the location of the positive and negative electrodes on the positions. Human, rabbit and guinea pig ECG have ST segment, however rat and mouse ECG have J wave. RA; right arm, LA; left arm, LL; left leg

**Fig. 4:**
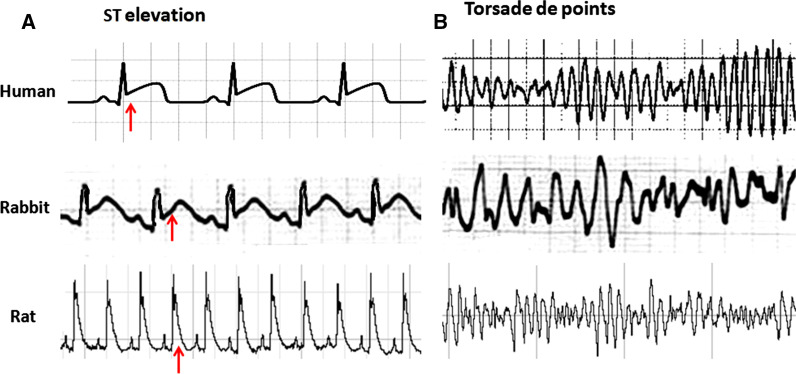
**A** ST segment elevation in human and rabbit ECG and J wave change in rat folowing to cardiac injury. **B** Ventricular torsade de points in human, rabbit and rat

Based on previous studies the approximate values of heart rate and some ECG parameters of human and other animals have been shown in Table 4 [57–65]. Also, the strips of Lead II ECG and the connection model of standard ECG leads in humans and rodents are demonstrated in Fig. 3. It is obviously a non isoelectric ST segment, but instead a characteristic J wave, the down-sloping portion of the ST segment that represents early repolarization, in ECG of rat and mouse. This pattern is due to a high transient outward current (Ito) of potassium in phase 1 repolarization. In addition, they usually show an ECG with a brief QT interval, the absence of a Q wave in most leads, the absence of clear separation between the QRS complex and the T and also the lack of a common isoelectric baseline for the P, QRS, and T waves (Fig. 3) [12]. In addition, usually the identity of the end of T wave in murine and hence estimation of QT interval and related disturbance and its interpretation is difficult. In humans, the end of ventricular activation corresponds to the end of the QRS complex. However, in mice, The QRS complex is not a good measure of total ventricular activation time and they show electromechanical uncoupling at rapid stimulation rates [46]. Interestingly, despite the dissimilarity in the pattern of potassium channels activation during repolarization, similar to species with isoelectric ST segment, perturbations such as cardiac injury produce a similar shift in the corresponding QRS-T wave region of the ECG in mice and rats. The type of torsade de pointes and other arrhythmias are similar to human and following to myocardial infarction and cardiomyopathy, ECG of rats show deep Q wave (Fig. 4) [66–69]. In addition, changes in rabbit and rat ECG parameters such as P-wave enlargement, prolonged PR and QT intervals, lengthening QRS, QRS axis deviation, and T-wave changes have predictive value for many forms of heart disturbance [12]. Previous studies confirmed that similar to humans, long QT intervals in rats may be associated with increasing ventricular mass and hypertrophy which leads to long duration of action potential [12]. The ECG changes in rats following many drugs over doses such as some tranquilizers, antidepressants, vasodilators and adrenergic drugs are similar to humans. However, rat’s ECG is resistant to cardiac glycoside digitalis [12]. In addition, given the different pattern of potassium channels activation during repolarization in humans and murine, to induction and study torsade de pointes (TSD) arrhythmia, a polymorphic VT in which the ventricular complex has peaks that seems to ‘twist’ around the isoelectric baseline, rat and mouse should be used with caution. In this case, the rabbit is the preferred for human surrogate.

### Cardiac channelopathies and animal models

Ion channels dysfunction due to either genetic or acquired factors cause diseases that are named cardiac channelopathies. The main arrhythmia syndromes stem from channelopathies in human are congenital long QT syndrome (LQTS), short QT syndrome (SQTS), Brugada syndrome (BrS), catecholaminergic polymorphic ventricular tachycardia (CPVT), familial lone atrial fibrillation (AF), familial bradycardia and variable types of conduction defects (CD). In this regard, some animal models especially mouse and rabbit models have been designed that have helped to develop the knowledge and attitude of scientists in the basic and clinical fields. Fortunately, for LQTS there are several models of rabbit as animal with most similarity action potential and ECG features with human. Transgenic rabbits with pore mutations in KCNQ1, KCNH2 and KCNE1 showed LQTS1, LQTS2 and LQTS5 phenotypes corresponding to their absence of IKs, IKr and IKs respectively [70, 71]. Also a rabbit model is designed with pore mutations in KCNH2 corresponding to increasing IKr current that mimic SQTS1 [72]. The genetic basis of some of inherited arrhythmias along with animals existing models are summarized in Table 5 [7, 9, 70–84].

**Table 5:** The genetic basis of some of inherited arrhythmias along with existing animals’ transgenic models

Arrhythmia syndrome	Current	Channel/protein	Gen	Functional Alteration	Reported Models	Ref
Familial atrial fibrillation	*I*Ks	Potassium channel (α-subunit)	KCNQ1	Gain of function	Mouse	[9, 76, 82, 83]
	*I*Kr	Potassium channel β-subunit	KCNE2	Gain of function	Mouse	[9, 76, 82, 83]
	*I*Kr	Potassium channel α-subunit	KCNH2	Gain of function		[9, 82, 83]
	*I*K1	Potassium channel subunit	KCNJ2	Gain of function		[9, 82, 83]
	IKUr	Potassium channel subunit	KCNA5	Loss of function		[81–83]
	*I*Na	Sodium channel α-subunit	SCN5A	Loss of function		[81–83]
Cardiac conduction disease	*I*Na	Sodium channel α-subunit	SCN5A	Loss of function	Mouse	[9, 73]
Sick sinus syndrome	*I*f	Pacemaker channel subunit	HCN4	Loss of function	Mouse	[7, 9, 50, 80]
	*I*Na	sodium channel α-subunit	SCN5A	Loss of function		[9, 80]
*Brugada syndromes*						
BrS1	*I*Na	Sodium channel α-subunit	SCN5A	Loss of function	Mouse, Porcine, Canine	[7, 9, 74, 80, 82]
BrS2	Regulation of INa	Glycerol-3-phosphate dehydrogenase 1-like	GPD1L	Altered function		[7, 9, 80, 82]
BrS3	*I*Ca	calcium channel α-subunit	CACNA1C	Loss of function		[7, 9, 78, 80, 82]
BrS4	*I*Ca	Calcium channel β-subunit	CACNB2b	Loss of function		[7, 9, 78, 80, 82]
*Long QT syndromes*						
LQTS1	*I*Ks	Potassium channel α-subunit	KCNQ1	Loss of function	Rabbit, mouse	[7, 9, 70, 71, 79, 80, 82]
LQTS2	*I*Kr	Potassium channel α-subunit	KCNH2	Loss of function	Rabbit, Mouse	[7, 9, 70, 71, 79, 80, 82]
LQTS3	*I*Na	Sodium channel α-subunit	SCN5A	Gain of function	Mouse	[7, 9, 79, 80, 82]
LQTS5	*I*Ks	Potassium channel β-subunit	KCNE1	Loss of function	Rabbit, Mouse	[7, 9, 70, 75, 79, 82]
LQTS6	*I*Kr	Potassium channel β-subunit	KCNE2	Loss of function		[7, 9, 79, 80]
LQTS7	*I*K1	Potassium channel subunit	KCNJ2	Loss of function	Mouse	[7, 9, 79, 80, 82]
LQTS8	ICa	Calcium channel α-subunit	CACNA1C	Gain of function		[7, 9, 78–80, 82]
*Short QT syndrome*						
SQTS1	*I*Kr	Potassium channel α-subunit	KCNH2	Gain of function	Rabbit	[7, 9, 72, 77, 80, 82]
SQTS2	*I*Ks	potassium channel α-subunit	KCNQ1	Gain of function		[7, 9, 77, 80, 82]
SQTS3	*I*K1	Potassium channel subunit	KCNJ2	Gain of function		[7, 9, 77, 80, 82]
SQTS4	ICa	Calcium channel α-subunit	CACNA1C	Loss of function		[77, 80]
SQTS5	ICa	Calcium channel β2-subunit	CACN2B	Loss of function		[77, 80]
SQTS6	ICa	Calcium channel α2δ1-subunit	CACNA2D1	Loss of function		[77, 80, 82]

LQTS, long QT syndrome; SQTS, short QT syndrome; BrS, Brugada syndrome; INa, Fast Na^+^ current; IKur, ultra-rapid outward K^+^ current; ICa, Ca^2+^ current; IKr, Rapid delayed rectifier K^+^ current; IKs, Slow delayed rectifier K^+^ current; IK1, inward rectifier potassium current

In addition to genetic manipulations, using pharmacological/chemical agents, burst pacing, ligation and ischemia/injury methods are other ways to induction of cardiac arrhythmia which their details are beyond the scope of this article. Also, some other factors such as the model of ischemia/injury induction, for example through drugs/agents or coronary ligation, and its severity, electrolytes levels especially serum potassium level, the amount and type of anesthetic drugs used, the amount and type of arrhythmogenic drugs used, heart rate and even blood pressure level can be affected on incidence and prevalence of arrhythmias [4]. For instance, severe injury and also high and low levels of potassium increase the arrhythmia susceptibility, however anesthetic agents such as halothane, xylazine and ketamine decrease the incidence of arrhythmia [4]. So, responsiveness to arrhythmogenic agents in conscious and unconscious conditions may be different and should be accounted for in animal studies. Previous studies reported that rats respond to antiarrhythmic agents class I, III and VI [12, 85]. Some studies indicate links between adverse cardiovascular outcomes and decreased HRV in rats and RR variability (RRV) and QT variability (QTV) provide complementary information in describing the functioning of vagal and sympathetic limbs of the autonomic nervous system in humans and rats [86].

## Conclusion

Electrophysiology and electrocardiography studies show that human action potential and ECG have some dissimilarity with rodents. The main differences are comes from the diversity in distribution and density of potassium channels and their opening pattern during the repolarization phase. IKto and IKur potassium channels have a dominant effect on ventricular repolarization in rats and mice, however, for humans, rabbits, and guinea pigs ventricular repolarization mostly relies on IKr and IKs potassium channels. This discrepancy results in J wave instead of ST segment and a vague T wave with no clear end in murine ECG. Therefore, human heart action potential and ECG has more similarity to rabbits and then to guinea pigs and thirdly to rats/mice. Differences in the type and pattern of activation of potassium channels during the development of action potential in rat/mouse with humans is the most important limitation of the use of murine models in the study of electrical properties of the heart. However, rat/mouse electrocardiography studies have been welcomed more and in comparison with other rodents still more common. There can be several reasons for this. First; rats and mice are smaller in size and have short gestation periods, easier breeding, lower cost, less individual differences, easier handling and more standardized. Second; large number of inbreed and different diseases and transgenic models of rats and mice are more available. Third; there are a greater number of species-specific reagents and research tools of rat/mouse in the market. Forth; alongside the above, the most important point is that many of the heart disorders presenting in the rat/mouse's ECG such as the effects of cardio- toxicants, cardiac ischemia/injury/necrosis, effects of other cardiac stressors, electrical conduction disturbance and most of different arrhythmias are generalizable to humans. Despite all of the above, ethical considerations and laboratory facilities are the two most important determinants of choosing the right species/model. Ethics dictate that fewer animals, preferably smaller rodents, be used in experimental studies. However, this study showed that rabbits and guinea pigs have priority over rats and mice for electrocardiogram studies. Overall, the results of this study suggest that with respect to ethical protocols, rabbit ion channels, action potential and ECG are more similar to humans and is the first choice for cardiac arrhythmia and ectrocardiographic studies, and the guinea pigs, rats and mice are placed in the next priorities, respectively. It should be noted that this prioritization can be helpful, but it does not detract from the value of electrocardiographic studies on different species and, depending on the circumstances, researchers can decide to select the appropriate species/model.

## Data Availability

Not applicable. This manuscript is a brief report which reviews previous scientific reports to provide a platform that can help researchers to identify the best species/model for heart researches whose studies results can be translated into clinical practice.

## Abbreviations

AF: Atrial fibrillation
AP: Action potential
ARP: Absolute refractory period
AVN: Atrioventricular node
BrS: Brugada syndrome
CD: Conduction defects
CPVT: Catecholaminergic polymorphic ventricular tachycardia
CVDs: Cardiovascular diseases
DADs: Delayed afterdepolarizations
EADs: Erly afterdepolarizations
ECG: Electrocardiogram
ERP: Effective refractory period
HCNC: Hyperpolarization-activated cyclic nucleotide-gated channel
HRV: Heart rate variability
ICaL: L-type Ca^2^^+^ current
IKr: Rapid delayed rectifier K^+^ current
IKs: Slow delayed rectifier K^+^ current
IK1: Inward rectifier potassium current
IKur: Ultra-rapid outward K^+^ current
INa: Fast Na^+^ current
INCX: Na^+^–Ca^2^^+^ exchanger current
Ist: Sustained inward Na^+^ current
LQTS: Long QT syndrome
MDA: Maximum diastolic potential
NCE: Na^+^–Ca^2^^+^ exchanger
RMP: Resting membrane potential
RRV: RR variability
SAN: Sinoatrial node
SQTS: Short QT syndrome
TRP: Transient receptor potential
TSD: Torsade de pointes
QTV: QT variability
